# Large-scale human myeloma cell line small molecule compound screen dataset

**DOI:** 10.1038/s41597-025-04989-8

**Published:** 2025-04-19

**Authors:** V. Keith Hughitt, John K. Simmons, Sayeh Gorjifard, Aleksandra Michalowski, Kelli Wilson, Xiaohu Zhang, Paul Shinn, Carleen Klumpp-Thomas, Crystal McKnight, Zina Itkin, Lu Chen, Sam Michael, Jonathan Keats, Craig Thomas, Beverly A. Mock

**Affiliations:** 1 Laboratory of Cancer Biology and Genetics, Center for Cancer Research, National Cancer Institute, Bethesda, MD USA; 2 Natera, San Carlos, CA USA; 3 University of Washington, Seattle, WA USA; 4 Chemical Genomics Center, Division of Preclinical Innovation, National Center for Advancing Translational Sciences, Bethesda, MD USA; 5 Translational Genomics Research Institute, Phoenix, AZ USA

**Keywords:** High-throughput screening, Myeloma

## Abstract

Multiple myeloma, a hematopoietic malignancy of terminally differentiated B cells, is the second most common hematological malignancy after leukemia. While patients have benefited from numerous advances in treatment in recent years resulting in significant increases to average survival time following diagnosis, myeloma remains incurable and relapse is common. To help identify novel therapeutic agents with efficacy against the disease and to search for biomarkers associated with differential response to treatment, a large-scale pharmacological screen was performed with 1,912 small molecule compounds tested at 11 doses for 47 human myeloma cell lines (HMCL). Raw and processed versions of the drug screen dataset are provided, as well as supportive information including drug and cell line metadata and high-level characterization of the most salient features of each. The dataset is publicly available at Zenodo and the workflow code used for data processing and generation of supporting figures and tables are available on GitHub.

## Background & Summary

Multiple myeloma is a malignancy of plasma cells which are terminally differentiated B cells. It is the second most common hematological malignancy with a global incidence estimated at 176,404^1^ cases and a relative 5-year survival rate estimated at 61.1%^2^. Copy number alterations and complex genomic rearrangements involving translocations of immunoglobulin enhancers to growth promoting genes are key drivers of malignancy^3^. Despite significant advancements in treatment and a corresponding increase in average expected survival time over the past several decades^4,5^, this cancer remains incurable with current treatment strategies.

High-throughput small molecule screening assays provide an effective means to test a large set of candidate compounds against multiple cell lines^6,7^. To help facilitate the discovery of novel compounds with therapeutic potential in multiple myeloma, a large-scale small molecule compound library containing 1,912 compounds (the NCATS Mechanism Interrogation PlatE, “MIPE” 4.0) was screened against a set of 47 diverse human multiple myeloma cell lines (HMCL). This dataset was mined to identify potential drug combinations and perform preclinical assessments of their utility as potential therapies for multiple myeloma^8^. Briefly, robust regression analyses were performed with 178 drugs that produced a curve class of −1.1 and had an AC50 less than 2 μM in at least 25 cell lines. From the 178 drugs, 43 combinations were highly correlated in their responses across the myeloma cell lines with r^2^ values > 0.5. A secondary screen was performed to find 6 combinations that simultaneously increased the levels of the tumor suppressor CDKN2A (p16) and decreased levels of the MYC oncogene. Among these 6 combinations, three were found to be highly synergistic in their action against several myeloma cell lines. Common genetic pathways up- and down-regulated involved the Tgfβ/Smad signaling and cell cycle transition pathways, respectively. The drug screen dataset includes many additional drugs not considered in that study. The goal of this data descriptor is to provide a clear description of the full drug screen dataset^9^ including additional supporting information, while also adjusting for spatial biases subsequently detected in the drug screen.

**Fig. 1:**
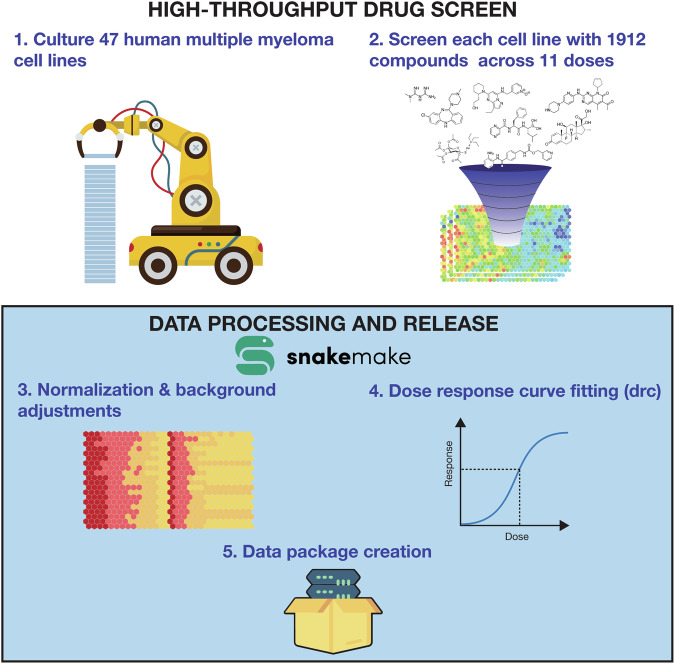
Experimental workflow. High-level overview of data generation and processing pipeline.

Raw and processed forms of the dataset are provided, as well as supporting metadata and a summary of the salient features present at the drug, cell line, and drug plate levels. An emphasis is placed on transparency and reproducibility, with the entire pipeline used to process the data and generate the tables and figures in this manuscript provided in the form of a Snakemake workflow (Fig. 1).

## Methods

### Cell culture

Human MM cells were cultured and authenticated as described previously^10^. Briefly, cells were cultured in RPMI 1640 with GlutaMAX L-glutamine (Life Technologies) supplemented with 10% fetal bovine serum (Cambrex BioScience), 100 U/ml penicillin and 100 μg/ml streptomycin (Life Technologies), prior to assay. Cell lines were provided by the Keats lab; sources and reference publications can be found at https://www.keatslab.org/myeloma-cell-lines/hmcl-public-availability.

### Small molecule screen

**Fig. 2:**
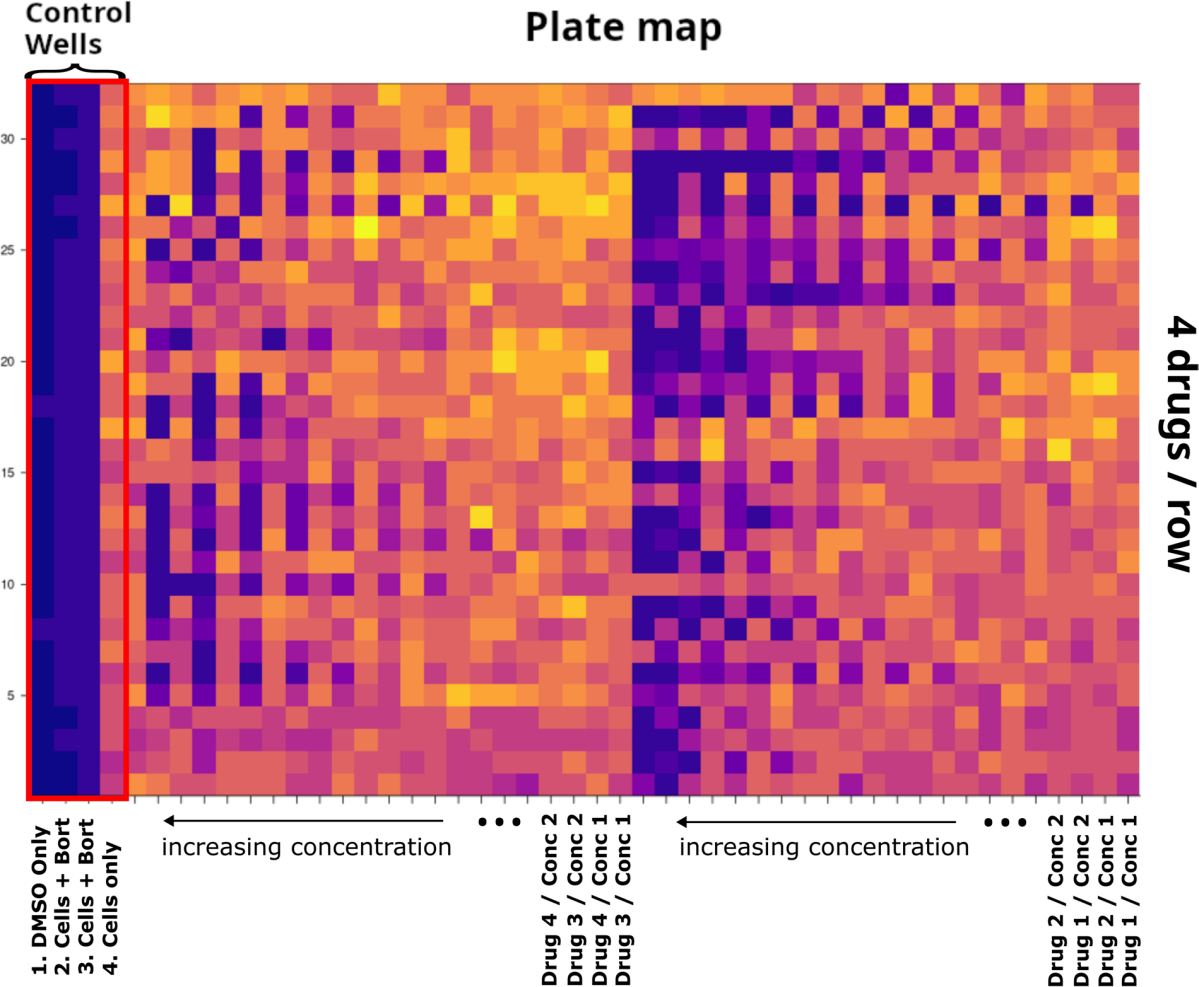
Plate map. Reference plate image depicting the locations of control wells and arrangement of treated wells for different drugs and concentrations. A raw L363 plate (T5612674) is used as an example.

The National Center for Advancing Translational Sciences MIPE (Mechanism Interrogation PlatE) compound library of 1,912 small molecules (“drugs/metadata.tsv” in the Zenodo dataset) was screened in 47 MM cell lines from the same passage of cells that had been sequenced previously (Table 1)^7^. Cell lines were treated in 1,536-well plates (Fig. 2) and the response was measured after 48-hour drug exposure with CellTiter-Glo® (Promega) luminescent cell viability assay at 11 doses (a serial of 3-fold dilutions), each in single replicate; https://ncats.nih.gov/research/research-activities/compound-management/chemical-libraries. As previously described^7^, a Multidrop Combi dispenser (Thermo Fisher) was used to dispense a total of 10^3 cells/well in 5μl of media (described above) into 1536 well solid-bottom white Greiner Bio-one tissue culture plates. A Kalypsys pintool was used to dispense 23 nl of control compound (bortezomib) and library compounds to generate the dose response curves. The 1536 well plates were covered with Kalypsys stainless steel cell culture lids and incubated at 37°C with 5% CO_2_ and 95% humidity. Cells were incubated for 48 h, after which 3 μl of CellTiter-Glo® was added with a Bioraptor Flying Reagent Dispenser (Aurora Discovery-BD) and incubated for 15 min at room temperature. The CellTiter-Glo signal was measured using a luminescent filter and 10 s exposure on a ViewLux (Perkin Elmer).

**Table 1:** Cell line metadata.

Cell Line	Sex	Ancestry	Clinical Heavy Chain	Clinical Light Chain	Canonical Translocations	KRAS	NRAS	TP53	TRAF3
AMO-1	Female	East Asia	IgA	Kappa	t(8;14)	A146T - het (cc)	Wt	Wt	A484Afs-het (cc)
ARD		Europe	IgA	Kappa	t(14;16)			HD	
JIM-1	Male	Europe	IgA	Lambda	t(4;14)			R273C - homo (cc)	
KHM-11	Male	East Asia	IgA	Kappa	t(4;14)	Wt	Q61K - het (cc)		Wt
KMM-1	Male	East Asia	Lambda	t(6;14) t(8;14)	Wt	G13D - homo (cc)	S241F - het (CCLE)(cc)	Wt	
KMS-12PE	Female	East Asia	Non-Secretory	Non-Secretory	t(11;14)	Wt	Wt	R337L - Homo (cc)	Wt
KMS-27	Male	East Asia	Kappa	t(11;14)		Q61 R - het (cc)	Splice-site_Splice-site (cc)		
Karpas 620	Female	Europe	IgG	Kappa	t(11;14)	G12D - het (cc)	Wt	C135Y - homo (CCLE)(cc)	Wt
L363	Female	Europe	IgG		t(20;22)	Wt	Q61H - Het (cc)	S261T - Homo	Wt
OCI-MY7		Europe			t(11;14)		Q61K - het (cc)		Wt
UTMC-2	Female	Europe	IgA	Kappa	t(4;14)	Wt	Wt	W246X - homo (cc) HD (?)	Wt
Delta47	Male	East Asia	IgD	Lambda	t(8;22)	Wt	Wt		Wt
H1112		Africa			t(11;14)	A146V - het (cc)	Wt	R248W - het (cc)	Wt
INA-6	Male	Europe	IgG	Kappa	t(11;14)		G12D - het (cc)	Q331X-het K132M-het(cc)	Wt
JIM-3	Male	Europe	IgA	Lambda	t(4;14)	G12D - het (cc)	Wt	R273C - homo (cc)	Wt
KMS-20	Female	East Asia	IgG	Kappa	t(8;14)	G12S - homo (cc)		Y126X - homo (CCLE)(cc)	Wt
Karpas 25	Female	Europe	IgG	Kappa	t(1;20)				
NCI-H929	Female	Europe	IgA	Kappa	t(4;14)	Wt	G13D - het (cc)	Wt	Wt
OPM-1	Female	East Asia	Lambda	t(4;14) t(8;14)	Q71X		R175H - homo (cc)	Wt	
PCM-6	Female	East Asia	IgG	Lambda	t(14;16)			R248Q (CCLE)	
PE2		Africa			t(4;14)		Q61K - het (cc)	G244V - homo (cc)	Wt
VP-6		Europe			t(14;16) t(8;14)			A161D - homo (cc)	
XG-1	Male	Europe	IgA	Kappa	t(11;14) t(8;14)	Wt	G12R - het (cc)	Y126N - homo (cc)	Wt
XG-6	Female	Europe	IgG	Lambda	t(16;22)	Q61E - het & L19F - het (cc)	Wt	Wt	Wt
ARP1		Europe	IgA	Kappa	t(14;16)			HD	
EJM	Female	Europe	IgG	Lambda	t(14;20)		Wt	K132N - homo (CCLE) (cc)	Wt
FR4	Male	East Asia	IgA	Kappa	t(8;14)	A146T - het (cc)	Wt	R175R - homo (cc)	Wt
JJN-3	Female	Europe	IgA1	Kappa	t(14;16) t(8;14)	Wt		HD (?)	Wt
KMS-12BM	Female	East Asia	Non-Secretory	Non-Secretory	t(11;14)	Wt	Wt	R337L - homo (cc)	Wt
KMS-26	Male	East Asia	IgG	Kappa	t(4;14) t(8;22)			R175H - homo (CCLE)(cc)	Wt
KMS-28PE	Female	East Asia	IgG	Lambda	t(4;14) t(8;14)	G12A - homo (cc)		G105R - homo (cc)	Wt
KMS-34	Female	East Asia	IgA	Kappa	t(4;14)			W146X - homo (CCLE)(cc)	S352N - het (cc)
LP-1	Female	Europe	IgG	Lambda	t(4;14) t(8;14)	Wt	Wt	E286K - Homo (cc)	K286IfsX7 - Homo (cc)
MM-M1		East Asia		t(11;14)		G12D - het (cc)		Wt	
MM.1R	Female	Africa	IgA	Lambda	t(14;16) t(8;14)	G12A - het (cc)			K536-N545delinsD-Homo (cc)
MM.1S	Female	Africa	IgA	Lambda	t(14;16) t(8;14)	G12A - het (cc)	Wt	Wt	K536-N545delinsD-Homo (cc)
MOLP-8		East Asia		t(11;14)	delK179 - het (cc)	Q61L - het (cc)			
OCI-MY1		Europe			t(8;22)	G13D - het (cc)		R280G - homo (cc)	HD
OCI-MY5		Europe			t(14;16) t(8;14)	Wt	Wt	FS - homo (cc)	Wt
OPM-2	Female	East Asia	Lambda	t(4;14) t(8;14)	Wt	Wt	R175H - homo (CCLE)(cc)	Wt	
RPMI-8226	Male	Africa	IgG	Lambda	t(16;22) t(8;22)	G12A - Het (cc)	Wt	E285K - Homo (cc)	HD - > K191LfsX60
SKMM-1	Male	Africa		Kappa	t(14;20) t(8;14)	Wt	G12D - het (cc)	R175G - het (cc)	Wt
U-266	Male	Europe	IgE	Lambda	t(11;14)	Wt	Wt	A161T - Homo (cc)	K550IfsX3 - Homo (cc)
KMS-11	Female	East Asia	IgG	Kappa	t(4;14) t(8;14) t(14;16)				
KMS-21BM	Male	East Asia	IgD	Lambda	t(11;14) t(8;14)		Q61L - het (cc)		
KMS-28BM	Female	East Asia	IgG	Lambda	t(4;14) t(8;14)	G12A - homo (cc)			Wt
Karpas 417	Male	Europe	IgG	Kappa	t(8;14)		Q61K - het (cc)		

Cell line metadata based on https://www.keatslab.org/myeloma-cell-lines/hmcl-characteristics.

HD - Homozygous Deletion, (cc) - Confirmed and/or Observed in the Cell line Characterization Project, (CCLE) - Broad Institute Cancer Cell Line Encyclopedia.

Plates from 4 of the 47 cell lines were found to deviate significantly due to potential issues with control wells (KMS11 & KMS28BM) or edge effects (KMS21BM & Karpas417) and were filtered out prior to background plate construction. Raw data for the outlier cell lines are provided.

### Normalization and background adjustment

Starting with the raw plate luminescence measurements, clipping was first applied within each plate, setting the small number of negative values present (<0.01%) to “0” and a maximum value equal to the 99.5% quantile. Normalized cell viability was computed as:

$${\rm{Viability}}\,( \% )=100\ast \,({\rm{well}}-{\rm{pos}})/({\rm{neg}}-{\rm{pos}})$$

Where “well” refers to the raw well luminescence values, and “pos” and “neg” refers to the median fluorescence measurements of the positive control wells (cells + bortezomib), and a negative control wells (cells + DMSO, dimethyl sulfoxide), respectively. Next, in order to adjust for spatial biases commonly encountered in high throughput screening assays^11^, a simple background adjustment approach was applied. A “background” plate matrix was constructed by taking the mean intensity for each well across all plates, and then subtracting the average within each specific drug concentration. The background plate was then subtracted from each raw plate matrix, and the resulting values were rescaled to the range [0, 100].

### Dose response curve fitting

For each cell and drug combination tested, four-parameter log-logistic dose-response models were fit using the drc package for R^12^. In cases where drc failed to converge, likely due to atypical dose response curves, the corresponding result entries were set to “NA”.

### Average cell line and drug behavior

**Fig. 3:**
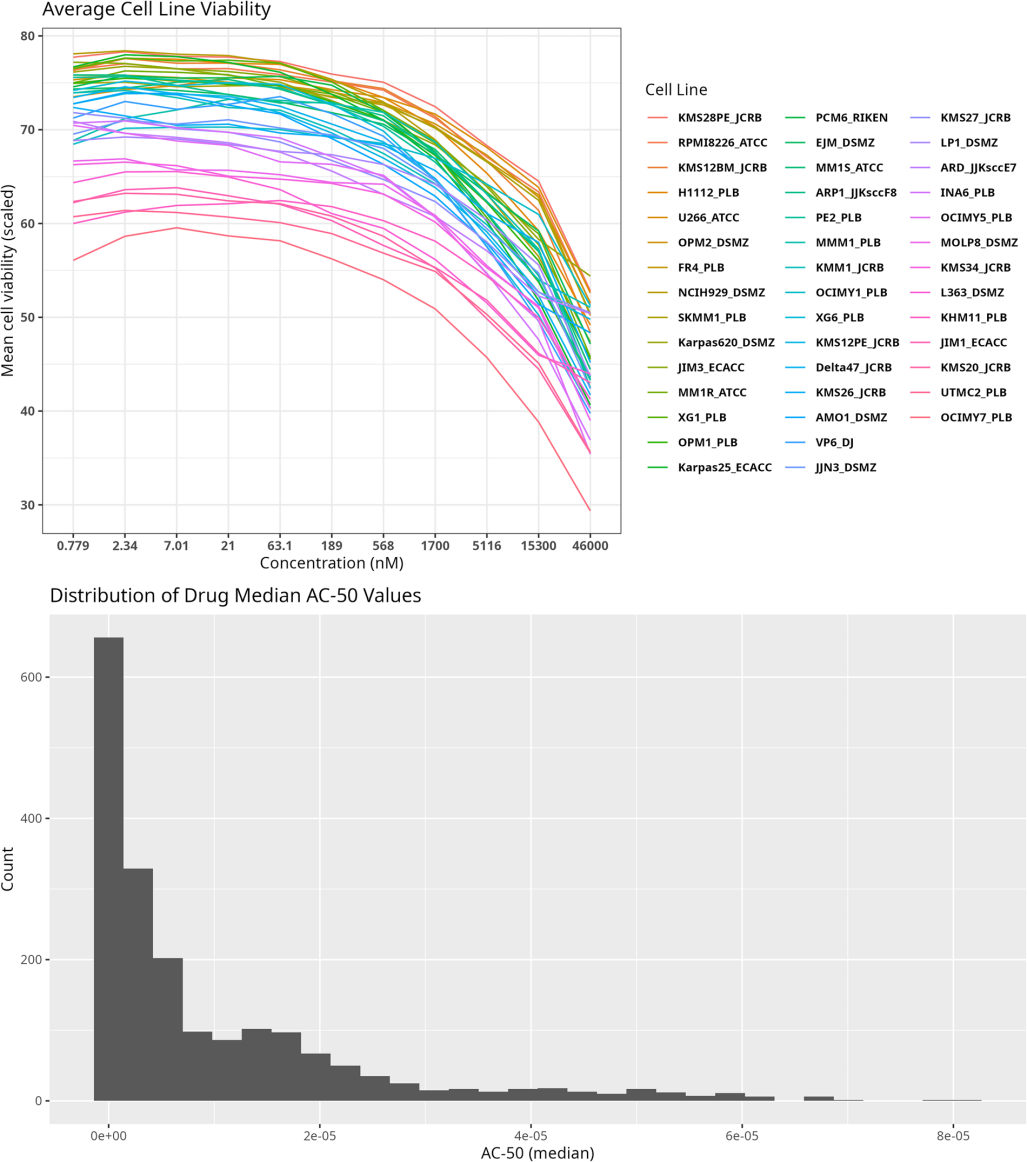
High-level view of differences in drug efficacy and cellular response to treatment. (**a**) For each cell line and dose increment, mean viability across all 1,912 compounds was computed and the resulting dose-response curves were plotted. (**b**) For each drug, median AC-50 values were computed and a histogram of the resulting averages was plotted.

For each cell line and each dosage increment, average viability was computed as the mean of the normalized viabilities across all 1,912 compounds tested. The resulting average response curves for each cell were combined into a single plot (Fig. 3a). Cells with higher average viability scores appear near the top, while those with lower average viability scores appear near the bottom of the plot. For each drug, median AC-50 values were computed, and the distribution of resulting AC-50 values visualized as a histogram (Fig. 3b, Table 2).

**Table 2:** Summary of drug behavior.

Inclusion Criteria	Number of drugs
i. <100 nM for> = 80% cell lines	36
ii. <500 nM for > = 80% cell lines, but >100 nM for one or more cell lines	60
iii. <1000 nM for > = 80% cell lines, but >500 nM for one or more cell lines	37
iv. >1000 nM for > = 80% cell lines	660
v. Remaining drugs	1119

The number of drugs with AC-50 values above/below one of several specified cutoffs (<100 nM, <500 nM, <1000 nM, and >1000 nM) for at least 80% of the cell lines was computed, and the resulting counts reported in Table 2.

### Computational pipeline

In order to facilitate transparency and reproducibility, a computational pipeline was created using the Snakemake workflow engine^13^, with code written primarily in the R programming language^14^. Figure 3 was generated by the pipeline using ggplot2^15^ with colorblind friendly palettes from ggpubfigs.^16^. Data generated by the pipeline was packaged into a data package^17^ with relevant metadata for each element included.

The workflow code used for data processing and generation of supporting figures and tables can be found at https://github.com/khughitt/hmcl-drug-screen-pipeline.

### Cell line mutations

Predicted mutations for 46/47 cell lines for which data are available is provided as a supporting table^9^. This is a modified version of the “HMCL69_Preliminary_Mutation_List” dataset available at https://www.keatslab.org/data-repository, with cell lines not present in the drug screen excluded and cell line identifiers harmonized. Briefly, the mutation calls originate from human whole exome data generated using the Agilent SureSelect V4 + UTR hybrid capture kit. Sequencing data was generated on an Illumina HiSeq 2000 using 83 × 83 paired-end sequencing. The data was aligned to GRCh37 using BWA followed by duplicate marking and base quality recalibration with GATK. Mutations were identified using SAMTOOLS and likely germline events were filtered out when seen in 1000 genomes, ExAC r0.3, or NHLBI esp6500si_v2_ssa137 and not seen in COSMIC v74. The consequences of the individual likely somatic mutations was determined with snpEFF.

These methods expand on descriptions from our previous work^8^.

## Data Records

Raw and processed versions of the drug screen data described in this data descriptor are available at Zenodo^9^. Processed drug screen data is provided in several different forms, facilitating comparisons between drugs, cell lines, and plates.

In addition to the drug screen data itself, drug and cell line metadata tables are provided, as well as all tables and figures generated in the computational pipeline described above.

Data is provided in the form of a Frictionless data package (https://datapackage.org/), organized in subfolders relating to workflow steps. A top-level “datapackage.yml” file provides machine-readable descriptions of the main data elements including basic information such as file checksums, field types, and descriptions of each table.

The raw screening data, including measurements for all cell lines assayed, can be found at raw/data.tsv. Each row in the table describes a single well measurement, with fields corresponding to cell line (cell_line), experiment date (date), plate identifier (plate), a software parameter which can be used as a proxy for technical batch (layer_name), well row and column numbers (row and col), unadjusted well value (well_value), drug identifier (sample_id), and drug concentration in moles (concentration).

The file “drugs/drug_curves.tsv” contains the model fit parameters (slope, lower_limit, upper_limit, ac50, and lac50) and P-values (ac50_pval) corresponding to the fitted dose response curves for each cell line + drug pair, as well as the drug concentrations (conc_0..conc_10) and adjusted viability measurements (dose_0..dose_10) used as inputs for model fitting. The file “drugs/ac50.tsv” contains a matrix of AC-50 values, with cell lines arranged along rows, and drugs along columns. In cases where the curve fitting algorithm failed to converge, missing values may be present, represented as “NA”. Drug metadata can be found in the file “drugs/metadata.tsv” and is based off of a table of drug information provided by NCATS for the MIPE 4.0 screen, with updates based on metadata for the more recent MIPE 6.0 screen.

The file “manuscript/tables/Table 1.tsv” contains cell line metadata, and is the basis for Table 1 in the manuscript. The table is derived from a table of cell line information obtained from https://www.keatslab.org/myeloma-cell-lines/hmcl-characteristics, and has been modified to exclude cell lines not present in the drug screen and to harmonize identifiers. The folder “**manuscript/figures/**” contains the plots used to construct Fig. 3 in the manuscript, as output by the pipeline.

The “plates/” folder contains versions of the drug screen data at different stages of processing: “plates/raw.tsv”, “plates/raw_filtered.tsv”, “plates/normed.tsv”, and “plates/background adjusted.tsv”. In each case, data for each plate are stored in a single column, with the plate identifiers in the first row. The file “plates/concentrations.tsv” has the same structure, but contains the drug concentrations associated with each measurement. The file “plates/background.tsv” contains the background plate used to adjust for spatial biases, and mirrors the structure of a single 48 × 32 plate. Plate-level metadata including experiment date, cell line, and various quality assurance-related fields is provided at “plates/metadata.tsv”. The folder “plates/images/” contains image representations of each individual plate, as well as images for the “background” plate and average (“mean” and “median”) plates.

Predicted mutations for 46/47 cell lines for which data are available is provided as a supporting table at mutations/hmcl-predicted-mutations.tsv.

An earlier processed version of the drug screen dataset is also available on PubChem, with one record per cell line (e.g. https://pubchem.ncbi.nlm.nih.gov/bioassay/1918926). The data resource described here includes both the raw data and an alternative processing accounting for previously unappreciated spatial bias, as well as supporting datasets and workflow code.

## Technical Validation

### Drug screen validation and quality assurance

Each drug screen plate included four control columns which were used for quality assessment and normalization (Fig. 2). The first column contained only media (DMSO, dimethyl sulfoxide), the second and third columns contained cells treated with a proteasome inhibitor with known efficacy in myeloma cells (bortezomib), serving as a positive control, and the fourth column contained cells with DMSO, serving as a negative control. Each plate was assigned a heuristic quality score based on the median Pearson correlation of cell viability curves on the plate with a logistic curve representing an idealized sigmoid dose response curve, allowing potentially problematic plates to be flagged.

Plates from one experimental date and two cell lines (MM1S_ATCC and U266_ATCC) were found to have issues with the control wells and were flagged in the plate metadata table. Four additional cell lines (Karpas417, KMS11, KMS21BM, and KMS28BM) were found to have more widespread unexpected plate behavior, including visible edge effects, significantly lower correlations with other cell lines and lower average viabilities for nearly all doses. These were flagged as “outlier cell lines” in the plate metadata table. Plate images were constructed for each level of processing and manually inspected, helping to identify potentially problematic plates and cell lines as well as spatial biases described above and in the “background adjustment” methods section. These four cell lines were excluded from all downstream processing and figure generation steps.

## Data Availability

All of the code used to process the raw drug screen dataset and to create the figures and tables described in this data descriptor are available as a Snakemake pipeline at: https://github.com/khughitt/hmcl-drug-screen-pipeline.

## References

[1] Huang, J. *et al*. The epidemiological landscape of multiple myeloma: a global cancer registry estimate of disease burden, risk factors, and temporal trends. *Lancet Haematol.* **9**, e670–e677 (2022). 35843248 10.1016/S2352-3026(22)00165-X

[2] Myeloma - Cancer Stat Facts. *SEER* https://seer.cancer.gov/statfacts/html/mulmy.html.

[3] Welsh, S. J. *et al*. Transcriptional heterogeneity overcomes super-enhancer disrupting drug combinations in multiple myeloma. *Blood Cancer Discov.* **5**, 34–55 (2023). 10.1158/2643-3230.BCD-23-0062 PMC10772542 37767768

[4] Meermeier, E. W., Bergsagel, P. L. & Chesi, M. Next-generation therapies for multiple myeloma. *Annu. Rev. Cancer Biol*. **8** (2024). 10.1146/annurev-cancerbio-061421-014236 PMC11449476 39364307

[5] Gulla, A. & Anderson, K. C. Multiple myeloma: the (r)evolution of current therapy and a glance into future. *Haematologica* **105**, 2358–2367 (2020). 33054076 10.3324/haematol.2020.247015 PMC7556665

[6] Huang, R. *et al*. The NCATS Pharmaceutical Collection: a 10-year update. *Drug Discov. Today* **24**, 2341–2349 (2019). 31585169 10.1016/j.drudis.2019.09.019

[7] Mathews Griner, L. A. *et al*. High-throughput combinatorial screening identifies drugs that cooperate with ibrutinib to kill activated B-cell-like diffuse large B-cell lymphoma cells. *Proc. Natl. Acad. Sci. USA.* **111**, 2349–2354 (2014). 24469833 10.1073/pnas.1311846111 PMC3926026

[8] Peat, T. J. *et al*. Drug combinations identified by high-throughput screening promote cell cycle transition and upregulate Smad pathways in myeloma. *Cancer Lett.* **568**, 216284 (2023). 37356470 10.1016/j.canlet.2023.216284 PMC10408729

[9] Hughitt, VK., Simmons, J., & Mock, B. Human Myeloma Cell Line (HMCL) NCATS MIPE 4.0 Drug Screen Dataset, *Zenodo*, 10.5281/zenodo.14902712 (2025).

[10] Simmons, J. K. *et al*. Cooperative Targets of Combined mTOR/HDAC Inhibition Promote MYC Degradation. *Mol. Cancer Ther.* **16**, 2008–2021 (2017). 28522584 10.1158/1535-7163.MCT-17-0171 PMC5587368

[11] Mazoure, B., Nadon, R. & Makarenkov, V. Identification and correction of spatial bias are essential for obtaining quality data in high-throughput screening technologies. *Sci. Rep.* **7**, 11921 (2017). 28931934 10.1038/s41598-017-11940-4 PMC5607347

[12] Ritz, C., Baty, F., Streibig, J. C. & Gerhard, D. Dose-Response Analysis Using R. *PLoS One* **10**, e0146021 (2015). 26717316 10.1371/journal.pone.0146021 PMC4696819

[13] Köster, J. & Rahmann, S. Snakemake—a scalable bioinformatics workflow engine. *Bioinformatics* **28**, 2520–2522 (2012). 22908215 10.1093/bioinformatics/bts480

[14] R Development Core Team, R. *R: A Language and Environment for Statistical Computing*. 10.1007/978-3-540-74686-7 (R Foundation for Statistical Computing, 2024).

[15] Wickham, H. *ggplot2*. (Springer New York, New York, NY, 2009).

[16] Steenwyk, J. L. & Rokas, A. Ggpubfigs: Colorblind-friendly color palettes and ggplot2 graphic system extensions for publication-quality scientific figures. *Microbiol. Resour. Announc.* **10**, e0087121 (2021). 34734767 10.1128/MRA.00871-21 PMC8567791

[17] Frictionless Data Package (v2). https://datapackage.org/standard/data-package/.

